# Toward a Transportable Cell Culture Platform for Evaluating Radiotherapy Dose Modifying Factors

**DOI:** 10.3390/ijms242115953

**Published:** 2023-11-03

**Authors:** Nicholas Carlson, Carrie D. House, Mauro Tambasco

**Affiliations:** 1 Department of Physics, San Diego State University, San Diego, CA 92182, USA; ncarlson8881@sdsu.edu; 2 Biology Department, San Diego State University, San Diego, CA 92182, USA; cdhouse@sdsu.edu

**Keywords:** cell shipping, dose modifying factor, oxygen enhancement ratio, cell irradiation, clonogenic assay, metabolic survival, transportable cell culture platform (TCCP), FLASH

## Abstract

The current tools for validating dose delivery and optimizing new radiotherapy technologies in radiation therapy do not account for important dose modifying factors (DMFs), such as variations in cellular repair capability, tumor oxygenation, ultra-high dose rates and the type of ionizing radiation used. These factors play a crucial role in tumor control and normal tissue complications. To address this need, we explored the feasibility of developing a transportable cell culture platform (TCCP) to assess the relative biological effectiveness (RBE) of ionizing radiation. We measured cell recovery, clonogenic viability and metabolic viability of MDA-MB-231 cells over several days at room temperature in a range of concentrations of fetal bovine serum (FBS) in medium-supplemented gelatin, under both normoxic and hypoxic oxygen environments. Additionally, we measured the clonogenic viability of the cells to characterize how the duration of the TCCP at room temperature affected their radiosensitivity at doses up to 16 Gy. We found that (78±2)% of MDA-MB-231 cells were successfully recovered after being kept at room temperature for three days in 50% FBS in medium-supplemented gelatin at hypoxia (0.4±0.1)% pO_2_, while metabolic and clonogenic viabilities as measured by ATP luminescence and colony formation were found to be (58±5)% and (57±4)%, respectively. Additionally, irradiating a TCCP under normoxic and hypoxic conditions yielded a clonogenic oxygen enhancement ratio (OER) of 1.4±0.6 and a metabolic OER of 1.9±0.4. Our results demonstrate that the TCCP can be used to assess the RBE of a DMF and provides a feasible platform for assessing DMFs in radiation therapy applications.

## 1. Introduction

Radiation therapy is a commonly used and effective treatment for loco-regional control of primary tumors [1]. The prescribed radiation dose is used as a surrogate measure of the biological effectiveness of the radiation [2]. However, the absorbed dose is a physical quantity (energy absorbed in a volume per unit mass of the volume), which does not always reflect the biological response of the tumor [3]. Various dose modifying factors (DMFs), such as radiation beam quality [4], dose rate (conventional versus ultra-high dose rate (FLASH)) [5] and tumor oxygenation [6], can result in diverse biological responses for the same absorbed dose delivered. For instance, extra care must be taken when using heavy ion therapy beams, such as protons or carbon ions, as their relative biological effectiveness (RBE) increases as the charged particle slows down [7], and this can impact normal tissues, which may receive the dose at the distal end of the tumor. Additionally, in the case of FLASH radiotherapy, physical parameters, such as total dose, dose rate, pulse rate (number and width), total beam duration, fractionation and radiation type [8,9], must be optimized for biological response and normal tissue sparing. Finally, tumor hypoxia will also affect the radiobiological response of low linear energy transfer (LET) radiation, as it makes tumor cells more resistant to the indirect action of low LET radiation on DNA damage.

**Figure 1:**
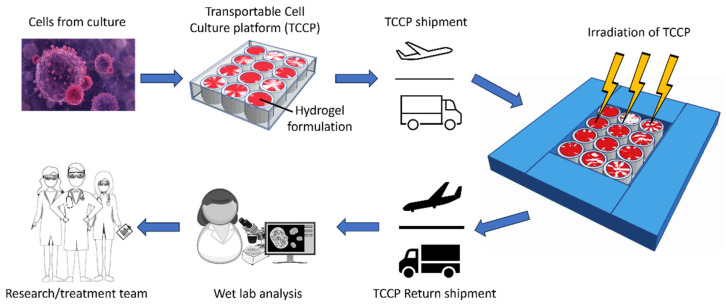
Proposed TCCP workflow. The TCCP is composed of cultured cells adhered to the bottom of a multi-well plate with gelatin media added over it to create a semi-solid gel state. The TCCP is shipped to the radiation site, where the TCCP can slot into a tissue-equivalent phantom cut-out to achieve a uniform radiation dose. After irradiation, the TCCP can be shipped back to the wet lab for analysis, and the results can be shared with the radiation research/treatment team.

Although medical physicists use physics-based dosimeters, such as ionization chambers, diodes, radiochromic film, etc., for quality control of the radiation beams and patient-specific doses, they do not possess adequate tools to evaluate DMFs, which can alter the RBE of a prescribed dose. Hence, there is a need for a biologically based platform to quantify and validate the relative radiobiological response of a given radiotherapy technology or patient-specific treatment. However, a significant limitation for medical physicists in utilizing such biologically based platform is their lack of wet lab skills, a wet lab facility and the equipment needed to perform biological analysis. Hence, we propose a transportable cell culture platform (TCCP), which serves as a biological tool for testing radiobiological responses under various clinical and research scenarios. Figure 1 shows the proposed workflow for TCCPs.

In this study, we construct and evaluate the ability of a TCCP to maintain sufficient cell viability at ambient room temperature for a few days to evaluate shippability to a clinical facility and back for the evaluation of radiation DMFs. As proof of principle, we used the metastatic MDA-MB-231 breast cancer cell line to demonstrate how the effects of hypoxia during radiation exposure can be evaluated with a TCCP.

Due to the high proliferation rate of cancer cells, tumor growth is often accompanied by regions, which are insufficiently oxygenated [10]. These hypoxic regions are rarely seen in normal tissues, but it is estimated that more than 80% of tumors have regions with oxygen tensions <0.3% [11,12,13,14,15,16,17]. Hypoxic regions are relatively hostile, and they drive the evolution of cancer cells toward more aggressive and metastatic phenotypes. For example, it is known that tumors with moderate to severe hypoxic regions are more resistant to treatment with radiation and/or chemotherapy [18,19,20]. Furthermore, the severity and extent of hypoxia in tumors have been found to correlate with poorer loco-regional tumor control [14,21], greater risk of metastasis [22,23] and poorer patient outcome [24]. Hence, hypoxia is a significant DMF to consider in cancer treatment.

Currently, a common approach for shipping cells to another facility involves the addition of a cryoprotectant called DMSO, followed by cell freezing and the use of dry ice to maintain the frozen state during transportation [25,26,27,28]. This approach is both expensive and challenging due to the strict shipping requirements for dry ice. Furthermore, it carries the risk of compromising cell viability if the shipment is delayed and the dry ice melts, as DMSO is toxic to cells [29]. Additionally, in the context of studying cellular radiation effects, the cells would have to be thawed for irradiation and frozen again for transport to a wet lab for analysis, which would further degrade cell viability. Hence, there is a clear need for a simpler, more cost-effective method of transporting cells for characterization of radiation DMFs.

Several methods of shipping cells at room temperature for the purpose of sharing them between distant research labs have been reported in the literature. These include shipping them in complete mixtures of semi-solid hydrogels, such as medium-supplemented Matrigel^®^ [30], a low-melting-temperature (LMT) agarose [31], a proprietary HemSol^TM^ gelatin mixture [32] and a medium-supplemented gelatin mixture [33]. In this study, we chose to test the use of a gelatin medium simply because it is three orders of magnitude cheaper than Matrigel^®^; it is commercially available, easily maintained in a solid gel form for irradiation at room temperature and easy to return to liquid state to recover cells for post-irradiation analysis. We initially explored the use of an LMT agarose, but we found that the melting temperature was too high (approximately 40 °C), which could potentially cause heat shock to the cells when melting the gel [34].

While it was reported by Ohyabu et al. [33] that cells transported with medium-supplemented gelatin maintained a viability of over 96% after 7 days at room temperature, as determined with trypan blue staining, trypan blue is unreliable for assessing viability below 80% [35], leading to possible overestimation of viability [36]. Furthermore, trypan blue only assesses viability due to membrane integrity and not metabolic and/or reproductive viability. Because the reproductive viability of cancer cells is a critical determinant of local tumor recurrence after radiotherapy treatment, we used the gold standard clonogenic assay in this study to assess the reproductive viability of the MDA-MB-231 cancer cells after a period of 1–7 days at room temperature in the TCCP. We also assessed metabolic viability via the amount of ATP present. Finally, as proof of principle for the use of the TCCP to quantify a DMF in radiotherapy, we also investigated the ability of the TCCP to assess the effect of hypoxia on cell viability.

## 2. Results

### 2.1. Survival of Cells at Room Temperature in the TCCP Hydrogel Formulation

**Figure 2:**
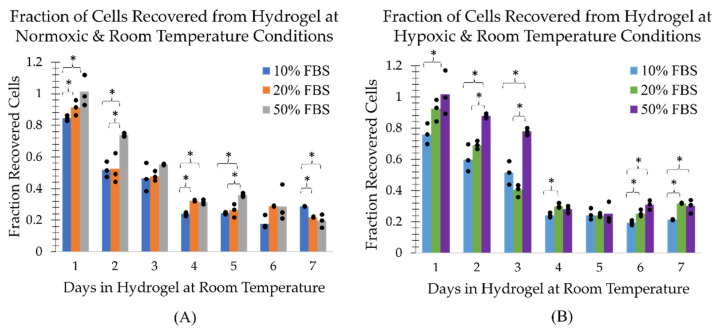
The fraction of viable cells as determined by trypan blue exclusion and a manual hemocytometer as a function of FBS hydrogel formulation concentration and days the TCCP was kept at room temperature. The fraction of recovered cells is shown for TCCPs kept under (**A**) normoxic and (**B**) hypoxic conditions. The three measurement points associated with each bar graph are plotted. An asterisk above a bracket covering all three bar graphs end to end indicates a statistically significant difference (*p* < 0.05) between all three groups. An asterisk above a bracket whose ends lie above the midpoints of two bar graphs indicates a statistically significant difference (*p* < 0.05) between only the two corresponding groups.

**Figure 3:**
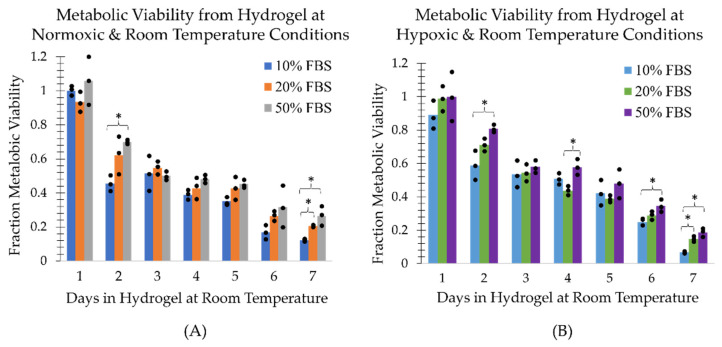
Metabolic viability as a function of FBS concentration and days the TCCP was kept at room temperature assessed with the CellTiter-GLO 2.0 assay and a subsequent 5-day incubation post-recovery. Metabolically viable cells normalized to day zero of the respective FBS concentration are shown for TCCPs kept under (**A**) normoxic and (**B**) hypoxic conditions. The three measurement points associated with each bar graph are plotted. An asterisk above a bracket covering all three bar graphs end to end indicates a statistically significant difference (*p* < 0.05) between all three groups. An asterisk above a bracket whose ends lie above the midpoints of two bar graphs indicates a statistically significant difference (*p* < 0.05) between only the two corresponding groups.

**Figure 4:**
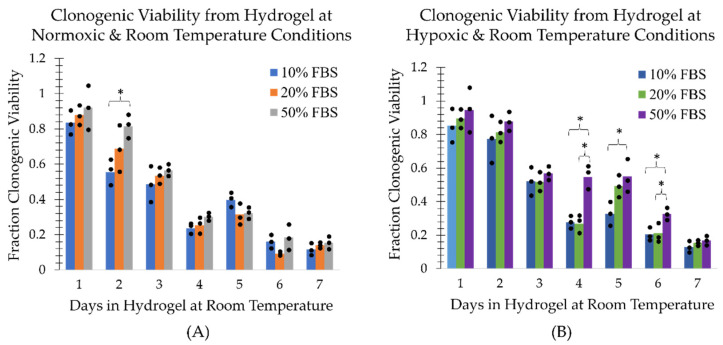
Fraction of clonogenic viability as a function of FBS concentration and days the TCCP was kept at ambient room temperature. The fraction of recovered cells is shown for TCCPs kept under (**A**) normoxic and (**B**) hypoxic conditions. The three measurement points associated with each bar graph are plotted. An asterisk above a bracket covering all three bar graphs end to end indicates a statistically significant difference (*p* < 0.05) between all three groups. An asterisk above a bracket whose ends lie above the midpoints of two bar graphs indicates a statistically significant difference (*p* < 0.05) between only the two corresponding groups.

The MDA-MB-231 cells in a mock shipment TCCP format at room temperature ((21.9±0.2) °C) in the normoxic environment (${\left(20.7\pm 0.3\right)\%\;pO}_{2})$ were found to have a recovery, metabolic and clonogenic viability half-life of approximately 3 days under normoxic conditions (Figure 2, Figure 3 and Figure 4, panel (A)). However, the TCCP cells in the hypoxic (${\left(0.4\pm 0.1\right)\%\;pO}_{2})$ environment had an increased metabolic and clonogenic half-life of up to 5 days (Figure 3 and Figure 4, panel (B)). The assays also generally showed an increase in viability as a function of FBS concentration (Figure 1, Figure 2 and Figure 3). Notably, within the initial 48 h, the 50% FBS hydrogel formulation concentration resulted in a (23±6)% increase in recovery from the normoxic environment (Figure 2A) and a (29±10)% increase in recovery from the hypoxic environment (Figure 2B) as compared to the 10% FBS hydrogel formulation. Similarly, the 50% FBS hydrogel formulation had (24±6)% and (22±10)% greater metabolic activity within the first 48 h in the normoxic (Figure 3A) and hypoxic (Figure 3B) environments, respectively, compared to the 10% FBS hydrogel formulation. After 48 h, the impact of FBS concentration on recovery and metabolic viability diminished, with less than a 10% difference observed, except for the outlier on day 3 in hypoxic recovery. However, the higher FBS concentration in the hydrogel formulation continued to show greater viability for the first 3 days in the normoxic environment (Figure 4A) and for up to 5 days in the hypoxic environment (Figure 4B).

**Figure 5:**
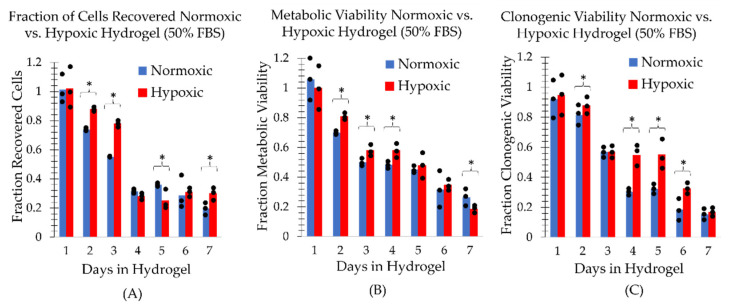
Comparison of hypoxic versus normoxic 50% FBS concentration TCCPs kept at ambient room temperature. The comparison is with respect to (**A**) recovered cells, (**B**) metabolic viability and (**C**) clonogenic viability. The three measurement points associated with each bar graph are plotted. An asterisk above a bracket covering the bar graphs indicates a statistically significant difference (*p* < 0.05) between all three groups.

Additionally, the MDA-MB-231 cells in hypoxic TCCPs generally showed greater cell recovery and metabolic and reproductive viability than cells in normoxic TCCPs (Figure 5). The impact of a hypoxic environment on TCCP cell viability was observed to have the most pronounced effect on cell recovery within the initial 3-day period, similar to the influence of FBS concentration. By day 3, hypoxia resulted in a (23±3)% increase in recovery compared to the normoxic environment (Figure 5A). The protective effect of hypoxia was also seen with respect to metabolic viability, which was greater than the normoxic TCCPs in the first 6 days (Figure 5B). However, the difference in metabolic viability between hypoxia and normoxia was minimal, with an increase of (11±4)% or less. The effect of hypoxia on clonogenic viability was minimal within the first 3 days, with a more prominent increase of (25±9)% and (23±13)% in reproductive viability on days 4 and 5, respectively (Figure 5C). Hence, the TCCPs composed of 50% FBS hydrogel formulation in a hypoxic environment at room temperature yielded the greatest cell recovery and metabolic and reproductive viability.

### 2.2. Recovery Time after Irradiation Affects Radiosensitivity of Cells in the TCCP

**Figure 6:**
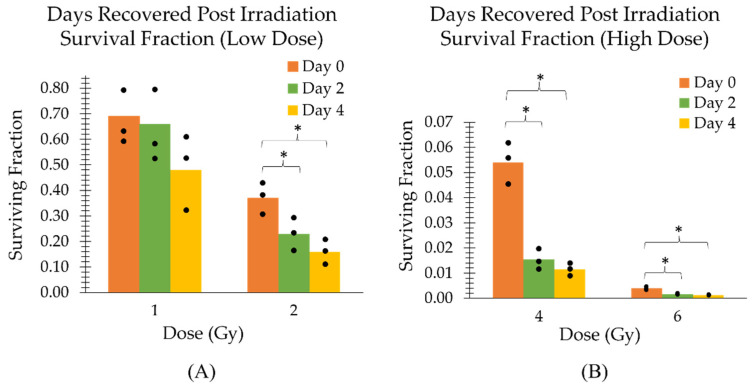
Surviving fractions are categorized by the number of days post-irradiation (**A**) low dose and (**B**) high dose, which the cells were recovered from the TCCPs kept at ambient room temperature. Day 0 is defined as recovery of the cells from the TCCP immediately after irradiation. The three measurement points associated with each bar graph are plotted. An asterisk above a bracket covering all three bar graphs end to end indicates a statistically significant difference (*p* < 0.05) between all three groups. An asterisk above a bracket whose ends lie above the midpoints of two bar graphs indicates a statistically significant difference (*p* < 0.05) between only the two corresponding groups.

The assessment of radiosensitivity using the clonogenic assay was affected by the suboptimal conditions of the cells in the TCCP. These conditions included a hypothermic ambient room temperature (21.9±0.2) °C and the absence of 5% CO_2_ supplementation to maintain the cells at an optimal physiological pH range. Figure 6 shows the effect on survival fraction when cells are kept in the TCCP at room temperature after irradiation.

The surviving fractions shown in Figure 6 for the MDA-MB-231 cells recovered immediately after irradiation (day 0) are in close agreement with those in the study by Abdullah et al. [37], which were 0.5, 0.2, 0.03 and 0.003, for 1, 2, 4 and 6 Gy, respectively. However, under the suboptimal room temperature conditions (days 2 and 4), the recovery time post-irradiation causes the cells to become radiosensitive and their survival fractions to decrease with each corresponding day (Figure 6). By day 2, while the survival fraction at 1 Gy compared to day 0 (recovered immediately after irradiation) is the same, the higher doses (2, 4 and 6 Gy) show a statistically significant decrease in cell survival (p<0.05). Additionally, by day 4 after irradiation, even a low dose of 1 Gy results in a (21±19)% decrease in survival.

### 2.3. Irradiated Hypoxic TCCPs Display a Relative Radiobiological Response

**Figure 7:**
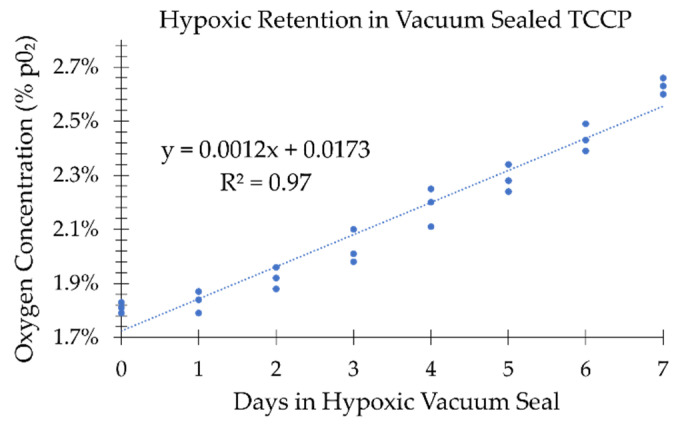
Oxygen concentration ($\%\;{pO}_{2}$) within the vacuum sealed hypoxic TCCP was monitored over 7 days in a normoxic $\left((20.7\pm 0.3)\%\;{pO}_{2}\right)$ ambient room temperature (21.9±0.2) °C environment. Measurements were taken after the vacuum sealed plate was removed from a hypoxic chamber after a 24 h equilibration period. The three measurement points are plotted for each day.

Figure 7 shows the ability of a vacuum seal bag to maintain a hypoxic environment for the TCCP. The $\%\;{pO}_{2}$ on day 0 at the time of removal from the hypoxic chamber was measured at $(1.81\pm 0.04)\%\;{pO}_{2}$, and on day 7, it was measured to be $(2.63\pm 0.02)\%\;{pO}_{2}$. Therefore, the vacuum seal only permitted an increase of $(0.82\pm 0.06)\%\;{pO}_{2}$ from day 0 to 7.

**Figure 8:**
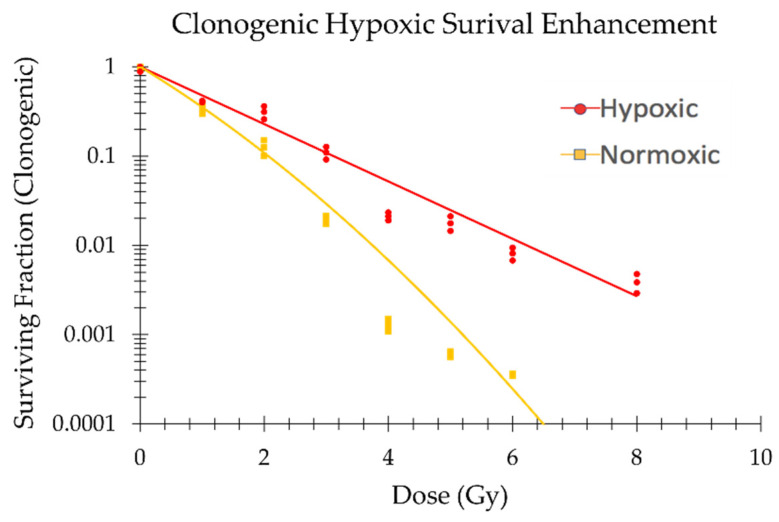
Clonogenic survival fractions and linear quadratic curves from irradiated hypoxic (($1.84\pm 0.03)\%\;pO_{2}$) and normoxic ($(20.7\pm 0.3)\%\;{pO}_{2}$) TCCPs. The three measurement points are plotted for each dose.

To observe a robust relative radiobiological response, characterized by hypoxic sparing and enhanced cell recovery and clonogenic viability, the TCCP plates were irradiated 24 h after being removed from the hypoxic chamber. This setup was designed to mimic an ideal overnight shipment, followed by irradiation the next day and an overnight return shipment, with recovery 24 h post-irradiation. At the time of irradiation, we measured the hypoxic TCCP samples to be $(1.84\pm 0.03)\%\;pO_{2}$, and they exhibited a value of $(1.92\pm 0.04)\%\;pO_{2}$ at the time of recovery. We measured the normoxic TCCP samples to be $(20.7\pm 0.3)\%\;pO_{2}$ at the time of irradiation and $(20.9\pm 0.4)\%\;pO_{2}$ at the time of recovery. Notably, we observed the hypoxic sparing effect when comparing hypoxic and normoxic TCCPs (Figure 8). The OER for the TCCPs was calculated at a 50% survival fraction, resulting in a value of 1.4±0.6. The α and β parameters constituting the linear quadratic curve fits (Equation (6)), as well as the OER values for normoxic and hypoxic TCCPs, are summarized in Table 1.

**Figure 9:**
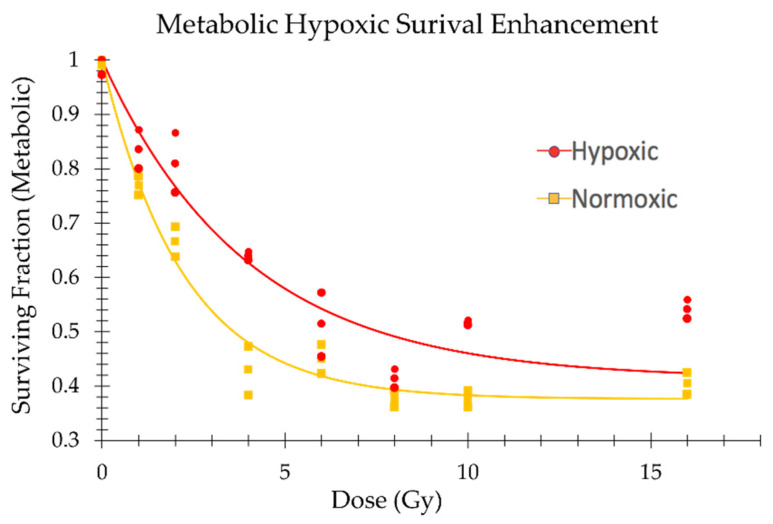
Metabolic survival fraction (MSF) under hypoxic and normoxic TCCP conditions from normalized RLU values using the CellTiter-GLO 2.0 assay to quantify the ATP present. The three measurement points are plotted for each dose.

The application of the CellTiter-GLO 2.0 assay to irradiated hypoxic and normoxic TCCPs to measure the fraction of cells which survived metabolically revealed a similar hypoxic sparing effect as the clonogenic assay (Figure 9).

After normalizing the relative light unit (RLU) values of the irradiated sample to the non-irradiated TCCP control, the data were found to follow an exponential decay with respect to dose, with a plateau in background RLU values at doses greater than 6 Gy. Consequently, the metabolic survival data exhibited a plateau-bounded exponential decay, which was fitted to the following equation:

(1) $$MSF=[\left(1-{SF}_{low}\right)\times e^{-\alpha D}]+{SF}_{low}$$

where MSF is the metabolic surviving fraction; *α* is the RLU decay constant; and ${SF}_{low}$ is the high dose background ATP signal. These parameters for the normoxic and hypoxic TCCPs are given in Table 2.

The metabolic OER (mOER) was computed using Equation (1) to find the ratio of the dose of the hypoxic TCCP to the normoxic TCCP to achieve an endpoint of 50% MSF. The MSF (50%) was as follows:

(2) $$\mathrm{MSF}(50\%)=1-\left(\frac{1-{SF}_{low}}{2}\right)=1-\left(\frac{1-0.40\;\pm \;0.01}{2}\right)=0.70\pm 0.01,$$

MSF(50%) from Equation (2) was used to look up the doses from Figure 9 that were used to compute the mOER. These doses and the mOER are given in Table 3.

## 3. Discussion

Currently, medical physicists optimize and perform quality control of novel radiotherapy technologies and patient-specific treatments using physics-based dosimeters. However, with the increasing use of heavy charged particle based therapies, which have high-end-track LETs, and the emergence of novel radiotherapy delivery technologies, such as FLASH, the absorbed dose is no longer always an adequate surrogate of the radiobiological effect. Hence, there is a growing need to develop a biologically based tool, which can be shipped to a radiotherapy facility, irradiated and shipped back to a wet lab for analysis without the use of dry ice.

This study aimed to evaluate the use of a cost-effective in-house hydrogel formulation platform for keeping cells alive at ambient room temperature in both normoxic and hypoxic environments to ultimately serve as a tool to evaluate the radiobiological effects of radiotherapy technologies and treatments. The hydrogel formulation was composed of lab-grade gelatin, FBS and RPMI nutritive media. The mixture was placed over the attached MDA-MB-231 cancer cells in a covered well plate to form the TCCP.

As proof of concept, we evaluated the ability of the TCCP to maintain cell viability at ambient room temperature and quantified the radiation dose modifying effect of hypoxia using the reproductive cell survival and metabolic ATP quantification assays. We chose the MDA-MB-231 cancer cell line because if the cell viability turned out to be insufficient for radiation studies with this hardy cell line, then the TCCP approach would have very little chance of working in less hardy cell lines.

Within the first 7 days, this study found that the 50% FBS hydrogel formulation outperformed the 10% and 20% FBS concentrations under both normoxic and hypoxic conditions. For example, within the first 48 h, 50% FBS compared to 10% FBS resulted in a (23±6)% increase in recovery, a (24±6)% increase in metabolic viability and a (26±14)% increase in clonogenic viability. In addition to the increased concentration of FBS, it was observed that a hypoxic versus normoxic TCCP environment contributed to  (32±11)% greater cell recovery and (15±14)% greater clonogenic viability on days 3 and 5, respectively. The ability of FBS to increase cell recovery and colony formation is consistent with other studies [39,40] due to its capacity for regulating cell growth, buffering pH changes [41] and maintaining osmotic pressure [42], which are all critical for cells at suboptimal conditions in the TCCP. The effects of lower oxygen concentrations on improved reproductive viability of 2D breast cancer cell line cultures have also been previously observed [43,44]. Hence, it is not surprising that the combination of increased FBS concentration and hypoxia yielded the greatest cell recovery and metabolic and reproductive cell viability.

Although these results using our gelatin formulation fall somewhat short of the viability reported by Wang, J. et al. using a Matrigel^®^ formulation [30], it is not clear whether this is due to a difference in the medium—i.e., their use of Dulbecco’s modified Eagles medium DMEM versus our use of RPMI—a difference in the hydrogel used, or a difference in the assay chosen to quantify viability. For example, Wang, J. et al.’s study did not quantify reproductive viability and used the MTT assay to assess metabolic viability, which can overestimate viability compared to the CellTiter-Glo^®^ metabolic assay [45] used in this study. Regarding the type of cell culture medium, this study’s findings differ from two previous studies, which used gelatin combined with DMEM [33] or HemSol^TM^ [32] across various cell lines, which reported greater viability. However, it is crucial to highlight that these studies assessed viability solely through the traditional trypan blue dye exclusion method. This method is less sensitive than metabolic assays and does not reflect reproductive viability, which was the principal assay employed in this study and is considered the gold standard for evaluating radiation toxicity. Future research is needed to assess the impact of hydrogel type and cell culture medium when using the same metabolic assay and clonogenic assay for reproductive cellular viability.

The observed decline in cell recovery in the TCCP with increasing time intervals between irradiation and cell recovery at higher doses is likely attributable to suboptimal hypothermia and a progressive decrease in pH. This pH reduction is a result of cells consuming nutrients and generating metabolites.

In this study, the OER at the 50% viability endpoint for the MDA-MB-231 cell line was found to be 1.4±0.6. This result is directionally in line with the higher OER of 1.8±0.7 reported by Lagadec et al., which was performed at a higher level of hypoxia (0.1% pO_2_) [43]. This expected directional agreement suggests that the TCCP is a reliable method for measuring dose modifying effects. Additionally, given the similarity of the OER with the mOER (1.9±0.5), the metabolic assay shows promise as a higher throughput assay than the clonogenic assay for evaluating a DMF. Moreover, the observation that CellTiter-Glo^®^ 2.0 was able to show hypoxic sparing at doses greater than 6 Gy is of interest. While the clonogenic assay could also be used to observe this effect, at doses above 6 Gy, this becomes challenging due to the large number of cells required. This is due to the reduced plating efficiency and radiosensitivity resulting from the time spent in the TCCP before recovery. In contrast, the metabolic survival assay only requires 1000 cells per well for each dose point, making it a potentially useful tool for quantifying DMFs at doses above 6 Gy. This includes the FLASH sparing effect, which is most prominent at doses above 10 Gy [46].

Overall, this study demonstrates the feasibility of using the TCCP to maintain cell viability at ambient room temperature long enough for one day shipping to a radiation therapy facility for irradiation and one day shipping back to a wet lab for analysis and evaluation of a DMF. However, the study has two limitations: (1) we only used one cell line; and (2) the results obtained with this hardy metastatic cell line may not extend to other cell lines or primary cancer cells.

In addition to the future studies suggested above, we plan to (1) investigate the robustness and reproducibility of using the TCCP to assess donor matched normal and tumor cell line viability in radiotherapy applications and to (2) construct a tissue-equivalent phantom to embed the TCCP for shipment to a radiotherapy clinic for irradiation and return shipment for analysis.

## 4. Materials and Methods

### 4.1. Cell Culture and Reagents

MDA-MB-231 breast cancer cells were cultured in RPMI 1640 medium with L-glutamine additives (Gibco^TM^, Thermo Fisher Scientific, Inc., Waltham, MA, USA) and supplemented with 10%, 20% or 50% fetal bovine serum (FBS) (Gibco^TM^, Thermo Fisher Scientific, Inc., Waltham, MA, USA) and 1% penicillin streptomycin (Gibco^TM^, Thermo Fisher Scientific, Inc., Waltham, MA, USA). Concentrations of FBS above the standard 10% were used to establish whether they could promote greater cell growth and viability. Cells were cultured in a humidified Galaxy 170S incubator (New Brunswick, Inc., Eppendorf AG, Germany) at 37 °C and 5% CO_2_.

### 4.2. Construction of the Cell Plate Platform

MDA-MB-231 cells in the liquid medium described in the next section were seeded into either a 12-well (Corning^TM^, Thermo Fisher Scientific, Inc.) or a 96-well white wall clear bottom (Stellar Scientific, Baltimore, MD, USA) culture plate and incubated at a density of $(2.5\;\pm \;0.2)\times 10^{5}$ cells/well or 1000±60 cells/well, respectively. They were stored in the incubator at 37 ℃ for 24 h to allow them to attach to the bottom of the wells. Once the cells were attached, the liquid culture media were removed and replaced with 2 mL or 200 mL (for 12-well or 96-well plate, respectively) of our hydrogel formulation (a mixture of RPMI 1640, FBS and 3% gelatin) designed to keep the cells viable at ambient room temperature. This hydrogel formulation, on top of the attached cells in the well culture plates, constituted the TCCP.

To assess the effects of hypoxia on the viability of the irradiated MDA-MB-231 cells, the TCCPs were prepared by placing them in a Torun AGB-3B vacuum glove box (Changshu Tongrun Electronic Co., Ltd., Changshu, China) filled with ultra-high-purity (99.999%) N_2_ gas and keeping them at ($0.4\pm 0.1)\%\;{pO}_{2}.$ The partial pressure of oxygen was measured with a Model 600 Oxygen Analyzer (Engineered Systems & Designs, Inc., Woburn, MA, USA). The TCCPs were left in the hypoxic chamber at ambient room temperature for a set number of days to mimic shipping time, with the plate lid left slightly ajar to ensure adequate nitrogen gas diffusion into the hydrogel formulation to achieve hypoxic equilibrium within the TCCP. Once the TCCPs were ready to be removed from the hypoxic chamber, they were vacuum sealed inside the hypoxic chamber with a vacuum sealer (DZ-290A Sinbo) in 3 mm thick transparent (combination of polyester and nylon) vacuum bags (CarePac, Inc., San Dimas, CA, USA).

Upon removal from the hypoxic chamber, the oxygen concentrations of surrogate water samples were measured with fiber-optic OXFOIL sensors using Pyroscience FireSting-pO_2_ (PyroScience, Inc., Aachen, Germany) equipment at the time of removal from the hypoxic chamber, at the time of irradiation and at the time of recovery. Surrogate water samples were used instead of gelatin medium, as the gelatin medium was found to interfere with the optical reading. That is, an equivalent volume of water was used in place of the gelatin medium. Additionally, the OXFOIL sensors were glued to the bottom of the well plate (corresponding to the location where the cells in the TCCP were attached) using acrylic transparent glue, so as not to interfere with the optical reading. The sensors were also calibrated to read through the transparent bottom of the well plate and the enclosing vacuum bag.

### 4.3. Procedure for Seeding and Recovering Cells from the TCCP Hydrogel Formulation

The following procedure was used to prepare the medium-supplemented gelatin solution (hydrogel formulation) of the TCCP:

1. Pre-warm a desired amount of 10–50% fetal bovine serum (FBS) supplemented RPMI complete growth medium to 37 ℃ in a bead bath.
2. Once it is warm, add HEPES (Gibco^TM^, Thermo Fisher Scientific, Inc.) pH buffer (pH 7.4) to a final concentration of 20 mM and mix using a vortex mixer (Fisher Scientific, Inc.) at 1500 rpm.
3. Add gelatin powder from bovine skin (Sigma-Aldrich, Inc., St. Louis, MO, USA) to the still 37 ℃ warm HEPES medium solution for a 3% final concentration of gelatin. Place back in the bead bath until all gelatin granules have melted and vortex again to ensure the solution is mixed thoroughly. Use immediately or store in the fridge for further use.
4. Add a working volume (i.e., 2 mL for 12-well plate or 200 μL for 96-well plate) of 37 ℃ liquid hydrogel formulation to adherent cells in a well plate by aspirating off the current liquid growth media and replacing them with gelatin medium.
5. Place the culture plate in a 4 ℃ fridge for 30 min for hydrogel formulation to set.
6. Remove the culture plate from the fridge and add parafilm between the plate lid and well rim to prevent evaporation of hydrogel formulation during transport.
7. To mimic ambient room temperature conditions, the hydrogel formulation TCCPs were left at 21.9±0.2 °C in a Styrofoam container on a lab bench for 1–7 days. After the allotted time, the TCCPs were recovered either following irradiation treatment to examine cell survival from irradiation or with no irradiation treatment to examine viability of the TCCP due to mock shipping time.

For recovery of TCCP cells

1. After the desired number of mock shipping days following irradiation or no irradiation treatment, the hydrogel formulation was removed by placing the TCCP plates in a 37 °C incubator for at least 30 min for the gelatin to melt.
2. After removing the hydrogel formulation, the cells were trypsinized using 0.25% trypsin (Gibco^TM^, Thermo Fisher Scientific, Inc.), counted with a Bright-Line hemocytometer (Hausser Scientific, Horsham, PA, USA) and diluted for clonogenic assay or given fresh complete RPMI growth medium for incubation in the CellTiter-Glo^®^ metabolic viability assay.

### 4.4. Fraction of Viable Cells Recovered

Immediately after the cells were trypsinized and collected from the TCCPs, they were pelleted with a 4×50 swing-bucket rotor centrifuge (Cole-Parmer, Inc., Vernon Hills, IL, USA) at 195 RCF and resuspended to a known volume of 0.50±0.06 mL of complete RPMI growth media. The concentration of viable cells recovered from the TCCP was assessed by applying the trypan blue (Gibco^TM^, Thermo Fisher Scientific, Inc.) stain and manually counting the cells with a Bright-Line hemocytometer (Hausser Scientific). This concentration was multiplied by the resuspended volume to estimate the number of viable cells recovered, and the fraction of recovered viable cells was computed using the following equation:

(3) $$\mathrm{Fraction}\;\mathrm{of}\;\mathrm{Viable}\;\mathrm{Cells}\;\mathrm{Recovered}=\frac{Number\;of\;viable\;cells\;recovered}{Original\;number\;of\;cells\;seeded\;in\;the\;CPP}$$

### 4.5. Radiation Treatment Protocol

The TCCPs were irradiated with either liquid medium or 3% gelatin medium covering them. The X-ray irradiation was delivered to the cells at a dose rate of 1.37±0.01 Gy/min, with 100 kVp and 5.0 mA delivery settings and a 0. 5 mm added aluminum filter using the CellRad irradiator (Faxitron, Tucson, AZ, USA). Samples were placed on the X-ray machine turntable at a distance of 23.0 cm from the X-ray source within a field size diameter of 16.2 cm. The turntable was set to rotate to ensure a uniform radiation dose coverage for the TCCPs. Beam flatness was measured by using Gafchromic EBT3 film (Ashland, Inc., Wilmington, DE, USA) and was found to be (6.0±0.8)% with the turntable rotation. Prior to the experiment, the CellRad system dose calibration was checked using the AAPM TG-61 protocol [47]. The TCCP cover and hydrogel formulation attenuation, the inverse square law and the shutter correction time (0.7 s) were computed and accounted for to ensure that the cells in the TCCP received the desired radiation dose.

### 4.6. Cell Survival Assay

Cells recovered from the TCCPs were trypsinized and seeded into 6-well plates in triplicate. Cell seeding numbers were dependent on the radiation dose received by the TCCP to account for decreased plating efficiency of cells, which spent a greater number of days at ambient room temperature. To allow for adequate colony growth, the seeded cells were incubated for 10–14 days, equivalent to 6–7 cell doublings for the MDA-MB-231 cell line. After incubation, the colonies were fixed with 100% methanol for at least 10 min and stained with 0.5% crystal violet (Innovating Science^®^ product from Aldon Corporation, Waltham, MA, USA) for at least 2 h. After washing, images of the colonies, defined as containing at least 50 cells, were manually counted using the FIJI (National Institutes of Health, Bethesda, MD, USA) cell counting feature, and the plating efficiency and survival fraction were calculated using Equations (4) and (5), respectively. Experimental survival fractions were fitted based on the linear quadratic function (Equation (6)) to determine the α  and β parameters using the Microsoft Excel evolutionary solver to find the least squares fit with the data (Microsoft Corporation, Redmond, WA, USA). Uncertainties in the α and β parameters were evaluated with non-linear parameter error propagation by evaluating the Hessian matrix. Clonogenic viability was normalized to plating efficiency at day 0 for the non-irradiated TCCPs (Equation (7)). Additionally, the OER between hypoxic and normoxic irradiated TCCPs was determined using Equation (8).

(4) $$\mathrm{Plating}\;\mathrm{Efficiency}=\frac{No.\;\;of\;colonies}{No.\;\;of\;cells\;plated}$$

(5) $$\mathrm{Irradiated}\;\mathrm{Surviving}\;\mathrm{Fraction}=\frac{No.\;\;of\;colonies}{No.\;\;of\;cells\;plated\times plating\;efficiency\;(0\;Gy)}$$

(6) $$\mathrm{Linear}\;\mathrm{Quadratic}\;\mathrm{Fit}=e^{-\alpha D-\beta D^{2}}$$

(7) $$\mathrm{Reproductive}\;\mathrm{Viability}=\frac{No.\;\;of\;colonies}{No.\;\;of\;cells\;plated\times plating\;efficiency\;(Day\;0)}$$

(8) $$\mathrm{OER}=\frac{Dose\;\left(Gy\right)\;hypoxic\;CPP}{Dose\;\left(Gy\right)\;normoxic\;CPP}\;\mathrm{at}\mathrm{the}\mathrm{same}\mathrm{surviving}\mathrm{fraction}.$$

### 4.7. ATP-Based Metabolic Viability Assay

Immediately after recovering the cells from the TCCPs, they were trypsinized and replated in triplicate into 96-well white walled clear bottom culture plates (Stellar Scientific, Inc.) at a density of 1000±60 cells/well.

After 6 days of cell incubation following recovery from the TCCPs, the fraction of metabolically viable cells was assessed by quantifying ATP using the CellTiter-Glo^®^ 2.0 luminescent assay (Promega, Inc., Madison, WI, USA). The relative light unit (RLU) luminescent signal was measured using a SpectraMax iD3 plate reader (Molecular Devices, LLC, San Jose, CA, USA). The survival fraction of the irradiated cells was found by normalizing the RLU raw signals for each radiation dose point to the 0 Gy control RLU raw signal (Equation (9)).

(9) $$\mathrm{Metabolic}\;\mathrm{Survival}\;\mathrm{Fraction}=\frac{RLU\;signal\;of\;irradiated\;CPP}{RLU\;signal\;of\;non-irradiated\;CPP\;(0\;Gy\;control)}$$

The metabolic viabilities of the non-irradiated cells recovered after being stored at ambient room temperature for a given number of days n were found by normalizing the RLU raw signal for day n in the TCCP to day 0 (cells removed from TCCP on the same day):

(10) $$\mathrm{Metabolic}\;\mathrm{Viability}=\frac{RLU\;signal\;(n\;days\;in\;the\;CPP)}{RLU\;signal\;(0\;days)}$$

### 4.8. Statistical Analysis

To assess the normality of each dataset, we used the Shapiro–Wilk test. For pairs of normally distributed datasets, we examined the equality of variances using the Levene non-parametric test. ANOVA was conducted to compare the means of the three datasets, which displayed both normality and homogeneity of variance. In cases where a trio dataset contained at least one set of data, which lacked both normality and homogeneity of variance, the Kruskal–Wallis non-parametric test was utilized. For pairs of datasets within the three groups, which passed the ANOVA or Kruskal–Wallis tests, we applied the Tukey test to find the means of the groups, which displayed a significant difference. For data where only two groups were compared, all the data satisfied the equality of variances, and a one-tailed pooled variance *t*-test was applied. All tests were conducted with a significance level of α<0.05.

## 5. Conclusions

We evaluated the feasibility of using a hydrogel formulation platform to transport cell cultures at ambient room temperature to assess the factors, which modify the relative amount of absorbed radiation dose required to achieve the same biological effectiveness. We used a metastatic breast cancer cell line (MDA-MB-231) and hypoxia as the dose modifying factor (DMF) to show the proof of concept for such platform. Further development of this platform could serve as a valuable tool for validating dose delivery and optimizing novel radiotherapy technologies in radiation therapy.

## Figures and Tables

**Table 1:** α and β parameters for hypoxic and normoxic TCCP clonogenic results, along with the corresponding α**/**β ratios. Uncertainties in the α and β parameters were computed using non-linear parameter error propagation by evaluating the Hessian matrix [38]. Additionally, the OER at a 50% survival rate is reported, including the propagated error. * Note: α/β for the hypoxic case is designated not applicable (NA) because the β parameter cannot be determined more accurately and this leads to a division by 0.

Condition	α	β	α /β	OER at 50% Survival
Normoxic	0.97±0.04	0.07±0.03	14±6	1.4±0.6
Hypoxic	0.7±0.1	0.00±0.05	NA *	

**Table 2:** *α* and ${SF}_{low}$ parameters for hypoxic versus normoxic TCCP metabolic results using the CellTiter-GLO 2.0 assay. Uncertainties in the *α* parameter were determined with non-linear parameter error propagation by evaluating the Hessian matrix [38].

Condition	α	*SF_low_*
Normoxic	0.45±0.04	(38±2)%
Hypoxic	0.25±0.04	(41±2)%

**Table 3:** Doses at MSF (50%) for hypoxic and normoxic metabolic TCCP and corresponding mOER.

Condition	Dose (Gy) at MSF (50%)	mOER at *MSF* (50%)
Normoxic	1.5±0.2	1.9± 0.4
Hypoxic	2.8±0.4	

## Data Availability

Please contact corresponding author (M.T.) for the raw data.

## References

[1] Baskar R., Lee K.A., Yeo R., Yeoh K.-W. (2012). Cancer and Radiation Therapy: Current Advances and Future Directions. Int. J. Med. Sci..

[2] Jones B., Dale R., Deehan C., Hopkins K., Morgan D. (2001). The Role of Biologically Effective Dose (BED) in Clinical Oncology. Clin. Oncol..

[3] Chang D.S., Lasley F.D., Das I.J., Mendonca M.S., Dynlacht J.R. (2014). Oxygen Effect, Relative Biological Effectiveness and Linear Energy Transfer. Basic Radiotherapy Physics and Biology.

[4] Kirkby K.J., Kirkby N.F., Burnet N.G., Owen H., Mackay R.I., Crellin A., Green S. (2020). Heavy charged particle beam therapy and related new radiotherapy technologies: The clinical potential, physics and technical developments required to deliver benefit for patients with cancer. Br. J. Radiol..

[5] Lin B., Gao F., Yang Y., Wu D., Zhang Y., Feng G., Dai T., Du X. (2021). FLASH Radiotherapy: History and Future. Front. Oncol..

[6] Wenzl T., Wilkens J.J. (2011). Theoretical analysis of the dose dependence of the oxygen enhancement ratio and its relevance for clinical applications. Radiat. Oncol..

[7] Underwood T.S., McMahon S.J. (2019). Proton Relative Biological Effectiveness (RBE): A Multiscale Problem. Br. J. Radiol..

[8] Bourhis J., Montay-Gruel P., Jorge P.G., Bailat C., Petit B., Ollivier J., Jeanneret-Sozzi W., Ozsahin M., Bochud F., Moeckli R. (2019). Clinical translation of FLASH radiotherapy: Why and how?. Radiother. Oncol..

[9] Petersson K. (2020). Collaborators FLASH radiotherapy: What, how and why?. Res. Outreach.

[10] Granchi C., Minutolo F. (2012). Anticancer Agents That Counteract Tumor Glycolysis. ChemMedChem.

[11] Rampling R., Cruickshank G., Lewis A.D., Fitzsimmons S.A., Workman P. (1994). Direct measurement of pO2 distribution and bioreductive enzymes in human malignant brain tumors. Int. J. Radiat. Oncol..

[12] E Lartigau E., Randrianarivelo H., Avril M.-F., A Margulis A., A Spatz A., Eschwége F., Guichard M. (1997). Intratumoral oxygen tension in metastatic melanoma. Melanoma Res..

[13] Nordsmark M., Alsner J., Keller J., Nielsen O.S., Jensen O.M., Horsman M.R., Overgaard J. (2001). Hypoxia in human soft tissue sarcomas: Adverse impact on survival and no association with p53 mutations. Br. J. Cancer.

[14] Movsas B., Chapman J.D., Hanlon A.L., Horwitz E.M., Pinover W.H., Greenberg R.E., Stobbe C., Hanks G.E. (2001). Hypoxia in Human Prostate Carcinoma: An Eppendorf Po_2_: Study. Am. J. Clin. Oncol..

[15] Fyles A., Milosevic M., Hedley D., Pintilie M., Levin W., Manchul L., Hill R.P. (2016). Tumor Hypoxia Has Independent Predictor Impact Only in Patients with Node-Negative Cervix Cancer. J. Clin. Oncol..

[16] Tomes L., Emberley E., Niu Y., Troup S., Pastorek J., Strange K., Harris A., Watson P.H. (2003). Necrosis and Hypoxia in Invasive Breast Carcinoma. Breast Cancer Res. Treat..

[17] Swinson D.E., Jones J.L., Richardson D., Wykoff C., Turley H., Pastorek J., Taub N., Harris A.L., O’byrne K.J. (2003). Carbonic Anhydrase IX Expression, a Novel Surrogate Marker of Tumor Hypoxia, Is Associated With a Poor Prognosis in Non–Small-Cell Lung Cancer. J. Clin. Oncol..

[18] Mottram J.C. (2014). A Factor of Importance in the Radio Sensitivity of Tumours. Br. J. Radiol..

[19] Gray L.H., Conger A.D., Ebert M., Hornsey S., Scott O.C.A. (2014). The Concentration of Oxygen Dissolved in Tissues at the Time of Irradiation as a Factor in Radiotherapy. Br. J. Radiol..

[20] Thomlinson R.H., Gray L.H. (1955). The Histological Structure of Some Human Lung Cancers and the Possible Implications for Radiotherapy. Br. J. Cancer.

[21] Nordsmark M., Overgaard J. (2009). Tumor hypoxia is independent of hemoglobin and prognostic for loco-regional tumor control after primary radiotherapy in advanced head and neck cancer. Acta Oncol..

[22] Rofstad E.K., Galappathi K., Mathiesen B., Ruud E.-B.M. (2007). Fluctuating and Diffusion-Limited Hypoxia in Hypoxia-Induced Metastasis. Clin. Cancer Res..

[23] Rischin D., Hicks R.J., Fisher R., Binns D., Corry J., Porceddu S., Peters L.J. (2006). Prognostic Significance of [^18^F]-Misonidazole Positron Emission Tomography–Detected Tumor Hypoxia in Patients with Advanced Head and Neck Cancer Randomly Assigned to Chemoradiation With or Without Tirapazamine: A Substudy of Trans-Tasman Radiation Oncology Group Study 98.02. J. Clin. Oncol..

[24] Vaupel P., Mayer A. (2007). Hypoxia in cancer: Significance and impact on clinical outcome. Cancer Metastasis Rev..

[25] Heydarzadeh S., Kia S.K., Boroomand S., Hedayati M. (2023). Recent Developments in Cell Line Shipping Methods: Pivotal Gaps in Cellular Death Sciences. Authorea Prepr..

[26] Sugawara Y., Sakai A. (1974). Survival of Suspension-cultured Sycamore Cells Cooled to the Temperature of Liquid Nitrogen. Plant Physiol..

[27] Hanna J., Hubel A. (2009). Preservation of stem cells. Organogenesis.

[28] Stevens V.L., Patel A.V., Feigelson H.S., Rodriguez C., Thun M.J., Calle E.E. (2007). Cryopreservation of Whole Blood Samples Collected in the Field for a Large Epidemiologic Study. Cancer Epidemiol. Biomarkers Prev..

[29] Chen X., Thibeault S. Effect of DMSO Concentration, Cell Density and Needle Gauge on the Viability of Cryopreserved Cells in Three Dimensional Hyaluronan Hydrogel. Proceedings of the 2013 35th Annual International Conference of the IEEE Engineering in Medicine and Biology Society (EMBC).

[30] Wang J., Chen P., Xu J., Zou J., Wang H., Chen H.-W. (2015). Transporting Cells in Semi-Solid Gel Condition and at Ambient Temperature. PLoS ONE.

[31] Wheatley S.P., Wheatley D.N. (2019). Transporting cells over several days without dry-ice. J. Cell Sci..

[32] Stefansson S., Han S., Jeon Y.I., Chung D.S., Hwang P., Le H., Warden J.L., Ho D. (2017). Transporting Mammalian Cells at Ambient Temperature: A Viable Alternative to Dry Ice. Adv. Biosci. Biotechnol..

[33] Ohyabu Y., Hatayama H., Yunoki S. (2014). Evaluation of gelatin hydrogel as a potential carrier for cell transportation. J. Biosci. Bioeng..

[34] Vogel P., Dux E., Wiessner C. (1997). Evidence of apoptosis in primary neuronal cultures after heat shock. Brain Res..

[35] Tennant J.R. (1964). Evaluation of the trypan blue technique for determination of cell viability. Transplantation.

[36] Chan L.L.-Y., Kuksin D., Laverty D.J., Saldi S., Qiu J. (2015). Morphological observation and analysis using automated image cytometry for the comparison of trypan blue and fluorescence-based viability detection method. Cytotechnology.

[37] Abdullah N., Inman M., Moody C.J., Storr S.J., Martin S.G. (2021). Cytotoxic and radiosensitising effects of a novel thioredoxin reductase inhibitor in breast cancer. Investig. New Drugs.

[38] Balsamo A., Mana G., Pennecchi F. (2006). The expression of uncertainty in non-linear parameter estimation. Metrologia.

[39] Fujisawa R., Mizuno M., Katano H., Otabe K., Ozeki N., Tsuji K., Koga H., Sekiya I. (2019). Cryopreservation in 95% serum with 5% DMSO maintains colony formation and chondrogenic abilities in human synovial mesenchymal stem cells. BMC Musculoskelet. Disord..

[40] Ye Y., Wang X., Ma C., Chen X., Liang H., Zhang W. (2020). Transporting ESCs in FBS at ambient temperature. Stem Cell Res..

[41] Chelladurai K.S., Christyraj J.D.S., Rajagopalan K., Yesudhason B.V., Venkatachalam S., Mohan M., Vasantha N.C., Christyraj J.R.S.S. (2021). Alternative to FBS in animal cell culture—An overview and future perspective. Heliyon.

[42] Weisberg H.F. (1978). Osmotic pressure of the serum proteins. Ann. Clin. Lab. Sci..

[43] Lagadec C., Dekmezian C., Bauché L., Pajonk F. (2012). Oxygen Levels Do Not Determine Radiation Survival of Breast Cancer Stem Cells. PLoS ONE.

[44] Timpano S., Guild B.D., Specker E.J., Melanson G., Medeiros P.J., Sproul S.L.J., Uniacke J. (2019). Physioxic human cell culture improves viability, metabolism, and mitochondrial morphology while reducing DNA damage. FASEB J..

[45] Malinowski P., Skała K., Jabłońska-Trypuć A., Koronkiewicz A., Wołejko E., Wydro U., Świderski G., Lewandowski W. (2022). Comparison of the Usefulness of MTT and CellTiterGlo Tests Applied for Cytotoxicity Evaluation of Compounds from the Group of Polyphenols. Environ. Sci. Proc..

[46] Böhlen T.T., Germond J.-F., Bourhis J., Vozenin M.-C., Ozsahin E.M., Bochud F., Bailat C., Moeckli R. (2022). Normal Tissue Sparing by FLASH as a Function of Single-Fraction Dose: A Quantitative Analysis. Int. J. Radiat. Oncol..

[47] Ma C.-M., Coffey C.W., DeWerd L.A., Liu C., Nath R., Seltzer S.M., Seuntjens J.P. (2001). AAPM protocol for 40-300 kV x-ray beam dosimetry in radiotherapy and radiobiology. Med. Phys..

